# A new species of *Raveniola* Zonstein, 1987 (Araneae, Nemesiidae) from Jinggangshan National Nature Reserve, Jiangxi Province, China

**DOI:** 10.3897/BDJ.14.e211761

**Published:** 2026-09-28

**Authors:** Congzheng Li, Qinlong Liang, Zhongjing Wang, Ke-Ke Liu

**Affiliations:** 1 Developmental Research Institute of Modern Agriculture, Jinggangshan University, Ji'an, China Developmental Research Institute of Modern Agriculture, Jinggangshan University Ji'an China; 2 College of Life Science, Jinggangshan University, Ji'an, China College of Life Science, Jinggangshan University Ji'an China

**Keywords:** East Asia, mygalomorph diversity, distribution, new record

## Abstract

**Background:**

Prior to this study, only two mygalomorph species from two distinct families had been formally recorded in Jinggangshan National Nature Reserve, Jiangxi Province: *Calommata jinggangica* Li & Xu, 2022 (Atypidae Thorell, 1870) and *Conothele jinggangshan* Liu, Xu, Zhang, Liu & Li, 2019 (Halonoproctidae Pocock, 1901), indicating that the overall mygalomorph diversity in this region remains far from fully revealed. The family Nemesiidae Simon, 1889, is known from the neighbouring provinces of Jiangxi: Hunan, Hubei, Fujian and Zhejiang. Nevertheless, no systematic surveys of Nemesiidae have ever been conducted in Jiangxi Province, and to date, no species belonging to this family has been formally reported from the region. During a recent exploratory field survey of local microhabitats in Jinggangshan National Nature Reserve, we collected a series of nemesiid specimens with distinctive morphology. Following morphological examination and preliminary molecular data, these specimens are confirmed to represent a new species of the genus *Raveniola* Zonstein, 1987. This discovery not only provides the first formal record of Nemesiidae from Jiangxi Province, but also highlights that the mygalomorph diversity in Jiangxi and eastern China remains largely under-investigated, with many potential taxa awaiting discovery.

**New information:**

The spider genus *Raveniola* Zonstein, 1987 is reported for the first time from Jiangxi Province, China, based on material collected in Jinggangshan National Nature Reserve. A new species, *R. zhangzongxuni* Liu, **sp. nov**., is described and illustrated. The discovery provides the first formal record of the family Nemesiidae from Jiangxi Province and bridges the known distributional records of the genus in eastern China, enhancing its geographic continuity on the map. The finding highlights that the mygalomorph diversity in Jiangxi and adjacent regions remains largely under-sampled and contributes to the understanding of the biogeography of the family Nemesiidae in East Asia.

## Introduction

The family Nemesiidae, belonging to the infraorder Mygalomorphae, comprises medium-sized to large burrowing spiders distributed only in the Northern Hemisphere. Amongst its constituent genera, *Raveniola* Zonstein, 1987 represents the most widely distributed genus in Asia, currently comprising 74 known species, with its range extending from West Asia through Central Asia to East Asia (Zonstein 1987, Zonstein et al. 2018, Zonstein 2024, World Spider Catalog 2026). The genus is diagnosed by the following combination of characters: ocular tubercle gradually elevated and conical, with the anterior median eyes distinctly raised; cheliceral furrow bearing a prolateral row of 8–12 homodont teeth and a few minute mesobasal denticles; maxillae with variable numbers of cuspules, ranging from a few to nearly one hundred, with significant interspecific variation; female spermathecae bifurcate, with the inner and outer branches frequently asymmetrical in shape, size and degree of sclerotisation; posterior median spinnerets often reduced to varying degrees or entirely absent; males with two (occasionally three) retroventral megaspines arranged sequentially on tibia I, which function to stabilise the female during copulation, his arrangement being shared amongst nemesiids only with *Sinopesa*, from which *Raveniola* differs primarily by having a noticeably longer and denser leg scopula (Zonstein et al. 2018, Zonstein 2024). Furthermore, species of *Raveniola* exhibit diverse habitat strategies: the majority utilise natural retreats such as cavities beneath stones or abandoned burrows of other animals as shelters, whereas only a minority inhabiting arid lowlands or high-altitude zones construct their own burrows (Zonstein 2024).

In China, the family Nemesiidae is currently represented by approximately 32 recorded species, belonging to four genera: *Damarchilus* Siliwal, Molur & Raven, 2015, *Nemesia* Audouin, 1826, *Raveniola* Zonstein, 1987 and *Sinopesa* Raven & Schwendinger, 1995 (World Spider Catalog 2026). Amongst these, *Raveniola* Zonstein, 1987 is the most species-rich genus, with known occurrences from the following provinces and municipalities: Yunnan, Guangxi, Sichuan, Fujian, Zhejiang, Hebei, Hubei, Tibet, Hunan, Shaanxi, Guangdong, Beijing and Henan (Li and Zonstein 2015, Tian et al. 2020, Lin et al. 2022, Lin et al. 2024, Zhou et al. 2025, Zonstein and Marusik 2025). According to the latest taxonomic study by Zonstein and Marusik (2025), all known Chinese *Raveniola* species have been preliminarily sorted into four distinct species groups (*R. alpina*, *R. beelzebub*, *R. gracilis*, *R. montana*) by the structural peculiarities of the male and female copulatory organs, the shape of the male tibia and metatarsus I or II, the number and arrangement of the maxillary or labial cuspules and the other structures. Only *R. xizangensis* cannot yet be definitively assigned to any established species group due to insufficient morphological data (Zonstein and Marusik 2025). Nevertheless, no species of Nemesiidae has ever been formally recorded from Jiangxi Province, reflecting that our knowledge of mygalomorph diversity in this region remains markedly insufficient.

During a recent exploratory field survey in Jinggangshan National Nature Reserve, Jiangxi Province, a series of nemesiid specimens was collected. Based on morphological examination and preliminary molecular data, these specimens are confirmed to represent a new species of the genus *Raveniola*. This discovery provides the first formal record of Nemesiidae from Jiangxi Province and extends the known distribution of the genus eastwards into central Jiangxi, thereby enriching the species diversity of *Raveniola* in eastern China and underscoring that the mygalomorph diversity of subtropical montane forests in this region remains far from fully explored.

## Materials and methods

Specimens were examined using a Jiangnan SZ6100 stereomicroscope. Male and female copulatory organs were dissected and examined in 80% ethanol. Epigynes were cleared with pancreatin solution (Álvarez-Padilla and Hormiga 2007). All specimens were photographed with a KUY NICE CCD camera, equipped with an Olympus CX43 compound microscope.

All measurements were made using a stereomicroscope (AxioVision SE64 rel. 4.8.3) and are given in millimetres. The total body length includes the chelicerae, but not the spinnerets. The length of the sternum was measured along the straight line between the posterior tip of the sternum and the hindmost part of the labium. Leg measurements are given as total length (femur, patella, tibia, metatarsus and tarsus). All examined specimens are deposited in the Animal Specimen Museum, College of Life Science, Jinggangshan University, Ji’an, China (ASM-JGSU).

DNA extraction, amplification and sequencing. DNA was isolated from legs (small size for right I−IV) and extractions performed with a Qiagen DNeasy Tissue Kit following a modified protocol of Valdez-Mondragón and Francke (2015). A DNA fragment corresponding to approximately 600 bp of the mitochondrial cytochrome coxidase subunit I (COI) gene was amplified using the same primers (LCO1490 and HCO2198) used by Valdez-Mondragón and Francke (2015). Amplifications were carried out using a Veriti 96 Well Thermal Cycler (Applied Biosystems) following a modified protocol of Valdez-Mondragón and Francke (2015), in a total PCR volume of 20 μl: 2.5 μl DNA, 2.3 μl H2O, 10 μl Multiplex-PCR Kit of Qiagen (which includes dNTPs, MgCl_2_, Taq PCR polymerase and Buffer), 3.2 μl of primers (1.6 of LCO1490, 1.6 of HCO2198) and 2 μl of Q-Solution (Qiagen). The PCR programme was as follows: initial step 15 min at 95°C; amplification 35 cycles of 35s at 94°C (denaturation), 1 min 30s at 40°C (annealing) and 1 min 30s at 72°C (elongation) and final elongation 10 min at 72°C (Valdez-Mondragón and Francke 2015).

Terminology of the male palp and female genitalia follows Zonstein (2024). The abbreviations used in the figures are as follows: ALE – anterior lateral eye, AME – anterior median eye, CB − copulatory bulb, Em – embolus, LD – lateral diverticulum, MOA – median ocular area, PLE – posterior lateral eye, PME – posterior median eye, SD – sperm duct, ST – spermathecal trunk.

## Taxon treatments

### Raveniola zhangzongxuni

Liu sp. nov.

B2797F30-1A73-554B-9CD8-3B924320EA52

76EBEC0F-80F1-4F28-9CD7-9DDF0AF0326F

#### Materials

- **Type status:** Holotype. **Occurrence:** catalogNumber: ASM-JGSU-Ara6960; recordedBy: Liu Ke-ke; individualCount: 1; sex: male; lifeStage: adult; occurrenceID: C34B8BE4-10E9-50B9-B3BE-A406C4A3679E; **Taxon:** scientificName: *Raveniola zhangzongxuni*; **Location:** country: China; stateProvince: Jiangxi; locality: Ji'an City, Jinggangshan County Level City, Jinggangshan National Nature Reserve, Jingzhushan; verbatimElevation: 1026.7 m; verbatimCoordinates: 26°32'28.34"N, 114°06'32.25"E; georeferenceProtocol: GPS; **Event:** samplingProtocol: handing; eventDate: 28/11/2025
- **Type status:** Paratype. **Occurrence:** catalogNumber: ASM-JGSU-Ara7438; recordedBy: Liu Ke-ke; individualCount: 1; sex: female; lifeStage: adult; occurrenceID: C6CB942A-EB5F-52A5-97F3-0038FEAAED03; **Taxon:** scientificName: *Raveniola zhangzongxuni*; **Location:** country: China; stateProvince: Jiangxi; locality: Ji'an City, Jinggangshan County Level City, Jinggangshan National Nature Reserve, Jingzhushan; verbatimElevation: 1026.7 m; verbatimCoordinates: 26°32'28.34"N, 114°06'32.25"E; georeferenceProtocol: GPS; **Event:** samplingProtocol: digging; eventDate: 28/11/2025
- **Type status:** Paratype. **Occurrence:** catalogNumber: ASM-JGSU-Ara6470; recordedBy: Liu Ke-ke; individualCount: 1; sex: female; lifeStage: adult; occurrenceID: AA47664F-C938-56AB-988C-D4459A6C593F; **Taxon:** scientificName: *Raveniola zhangzongxuni*; **Location:** country: China; stateProvince: Jiangxi; locality: Ji'an City, Jinggangshan County Level City, Jinggangshan National Nature Reserve, Lingxiufeng Scenic Spot; verbatimElevation: 945 m; verbatimCoordinates: 26°34'12.16"N, 114°07'15.21"E; georeferenceProtocol: GPS; **Event:** samplingProtocol: digging; eventDate: 04/10/2025
- **Type status:** Paratype. **Occurrence:** catalogNumber: ASM-JGSU-Ara9052; recordedBy: Liu Ke-ke; individualCount: 1; sex: female; lifeStage: adult; occurrenceID: 7BED0499-0BB6-5051-93B1-0A6829BBBF46; **Taxon:** scientificName: *Raveniola zhangzongxuni*; **Location:** country: China; stateProvince: Jiangxi; locality: Ji'an City, Jinggangshan County Level City, Jinggangshan National Nature Reserve, Zhufeng Scenic Spot; verbatimElevation: 867 m; verbatimCoordinates: 26°32'39.55"N, 114°08'52.54"E; georeferenceProtocol: GPS; **Event:** samplingProtocol: digging; eventDate: 29/11/2025

#### Description

**Figure 1.**
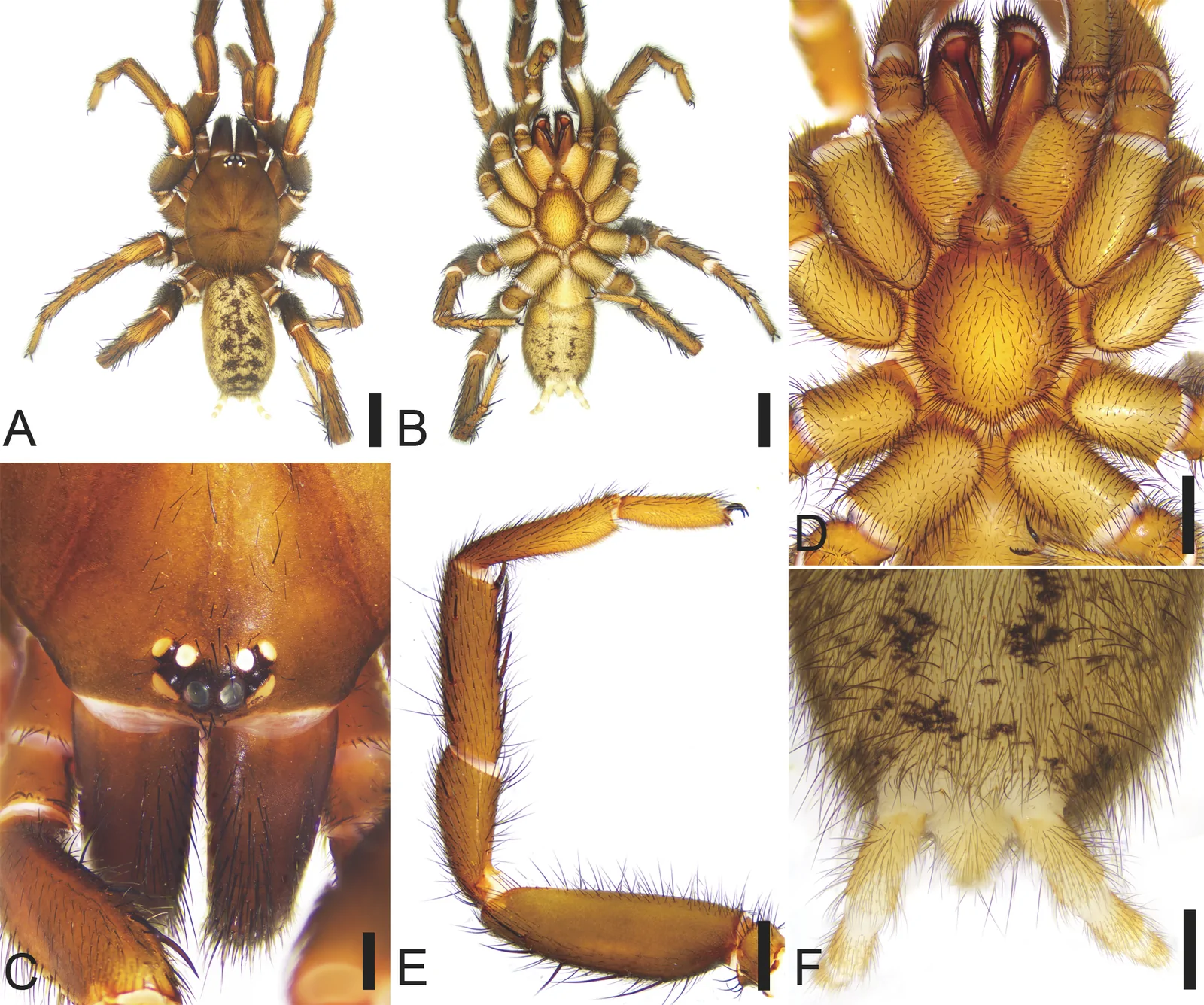
*Raveniola zhangzongxuni* sp. nov., male (holotype). **A** habitus, dorsal view; **B** same, ventral view; **C** eye tubercle and clypeus, dorsal view; **D** labium, maxillae and sternum, ventral view; **E** leg I, retrolateral views; **F** spinnerets, ventral view. Scale bars: 2 mm (A, B), 0.5 mm (C, D, F), 1 mm (E).

Male (holotype). Total length 10.94. General appearance as in (Figs 1, 4A−C) Carapace: 4.59 long, 3.66 wide, covered with few short black hairs. Eye diameters and interdistances: AME 0.15, ALE 0.22, PLE 0.14, PME 0.13, AME−AME 0.08, AME−ALE 0.09, ALE−PLE 0.10, AME−PME 0.08, PME−PME 0.27, PME−PLE 0.06. Each cheliceral furrow with 11 promarginal teeth and 9 mesobasal denticles. Maxillae 1.71 long, 0.93 wide, with 3 cuspules each. Labium 0.29 long, 0.77 wide. Sternum 2.33 long, 2.02 wide. Palp: 6.71 (2.40, 1.60, 1.87, −, 0.84). Leg I: 12.50 (3.63, 2.24, 2.75, 2.35, 1.53). Leg II: 11.22 (3.28, 1.96, 2.39, 2.24, 1.35). Leg III: 10.72 (2.87, 1.67, 2.03, 2.61 1.54). Leg IV: 14.80 (3.68, 2.03, 3.10, 4.07, 1.92). Leg formula: 4123. Leg I: tibia slightly incrassate, metatarsus slightly curved retroventrally.

Spination (Fig. 1A, B and E). Palp: Fe: d3, p1; Ti: p1, r1, v5; I: Fe: d4; Pa: p1; Ti: p3, v6; II: Fe: d3, p1; Ti: p4, v5; Mt: p1, v5; III: Fe: d8, p1; Pa: d3, p1; Ti: d4, p2, r3, v6; Mt: d7, p1, r1, v7; IV: Fe: d5, p3; Pa: p2, r1; Ti: d6, p3 ,r3 , v6; Mt: d3, p2, r1, v4.

Colouration in alcohol (Fig. 1): Carapace yellowish-brown, with radial dark brown marks; fovea distinct, red-brown; chelicerae reddish-brown; palps and legs reddish-brown to yellowish-brown; sternum, labium and maxillae yellowish-brown; abdomen dorsally light greyish-brown, with few brownish pattern transverse marks; ventral surface of abdomen light greyish-brown with few irregular brownish marks; spinnerets light yellowish-grey.

Palp (Fig. 3A−E). Patellar almost 2 times longer than wide, nearly as long as 2/3 tibia. Tibia almost 3.2 times as long as wide, about 2.5 times longer than cymbium, 2 thick spines located at distal part in prolateral view, 6 thick spines at ventral part (4 in ventro-prolateral and 2 in ventro-retrolateral aspects) in ventral view, one thick spine at distal part in retrolateral view. Cymbium oval, slightly less 1/2 as wide as long, also longer than 2/3 of copulatory bulb, dorsally with 5 thick spines. Copulatory bulb relatively broad, cone-shaped, 1.3 times as long as wide, with anticlockwise spiralling base. Sperm duct broad, transversally extending in retrolateral view, nearly covering 1/3 of copulatory bulb. Copulatory bulb + embolus longer than 1/2 tibia, more than 1.4 times longer than cymbium. Embolus slightly longer than copulatory bulb, with a curved apex forming an obtuse angle of about 115° in prolateral view.

Spinnerets (Fig. 1F). PMS absent. PLS: maximum diameter 0.45; length of basal, medial and apical segments 0.65, 0.57, 0.46; total length 1.68.

**Figure 2.**
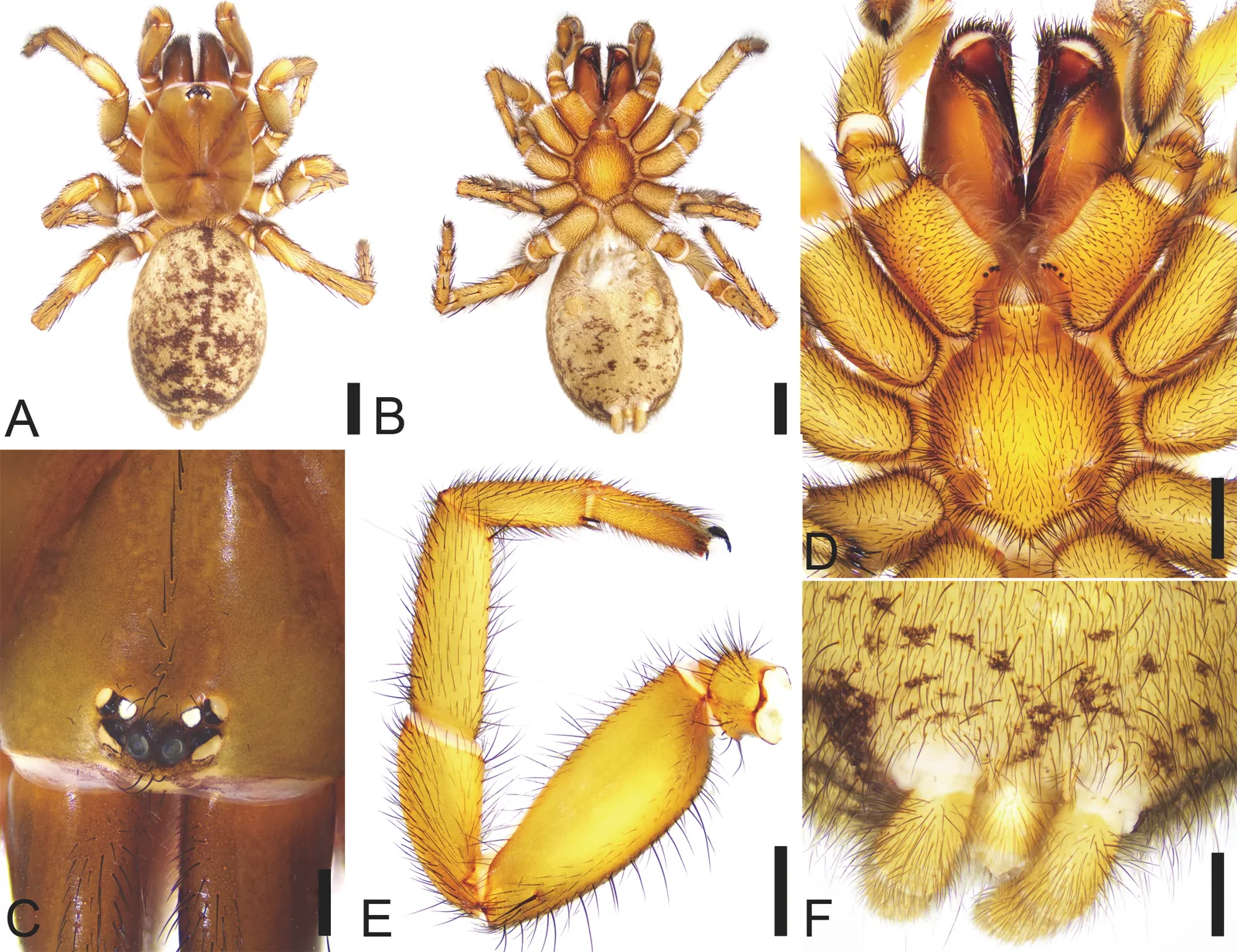
*Raveniola zhangzongxuni* sp. nov., female (paratype). **A** habitus, dorsal view; **B** same, ventral view; **C** eye tubercle and clypeus, dorsal view; **D** labium, maxillae and sternum, ventral view; **E** leg I, retrolateral views; **F** spinnerets, ventral view. Scale bars: 2 mm (A, B), 0.5 mm (C, F), 1 mm (D, E).

Female (Paratype, ASM-JGSU-Ara7438) (Figs 2, 4D−F). As in male, except as noted. Body length 14.61. Colour in alcohol as in male, but slightly paler. Carapace: 5.18 long, 4.20 wide. Eye diameters and interdistances: AME 0.17, ALE 0.27, PLE 0.18, PME 0.15, AME−AME 0.11, AME−ALE 0.09, ALE−PLE 0.12, AME−PME 0.13, PME−PME 0.36, PME−PLE 0.11. Each cheliceral furrow with 11 promarginal teeth and 8 mesobasal denticles. Maxillae 2.01 long, 1.08 wide, with 4 cuspules each. Labium 0.41 long, 0.83 wide. Sternum 2.46 long, 2.51 wide. Palp: 7.23 (2.45, 1.62, 1.78, −, 1.38). Leg I: 11.16 (3.36, 2.34, 2.49, 1.82, 1.19). Leg II: 10.51 (3.21, 2.16, 2.19, 1.72, 1.23). Leg III: 9.71 (2.84, 1.55, 1.84, 2.04 1.44). Leg IV: 14.67 (3.92, 1.96, 3.07, 2.87, 1.85). Leg formula: 4123.

Spination (Fig. 2A, B and E). Palp: Fe: d2, p1; Ti: v5; Ta: v3; I: Fe: p1; Ti: v1; Mt: 4v; II: Fe: d1, p1; Pa: d1; Ti: p2, v1; Mt: p1, v6; III: Fe: d3; Pa: d2, r1; Ti: d5, p1, r1, v6; Mt: d9, p1, v7; IV: Fe: d2; Pa: p2, r1; Ti: d7, p1, v6; Mt: d6, p3, r3, v7.

Spinnerets (Fig. 2F). PMS: length 0.26; diameter 0.09. PLS: maximum diameter 0.47; length of basal, medial and apical segments 0.78, 0.55, 0.52; total length 1.85.

**Figure 3.**
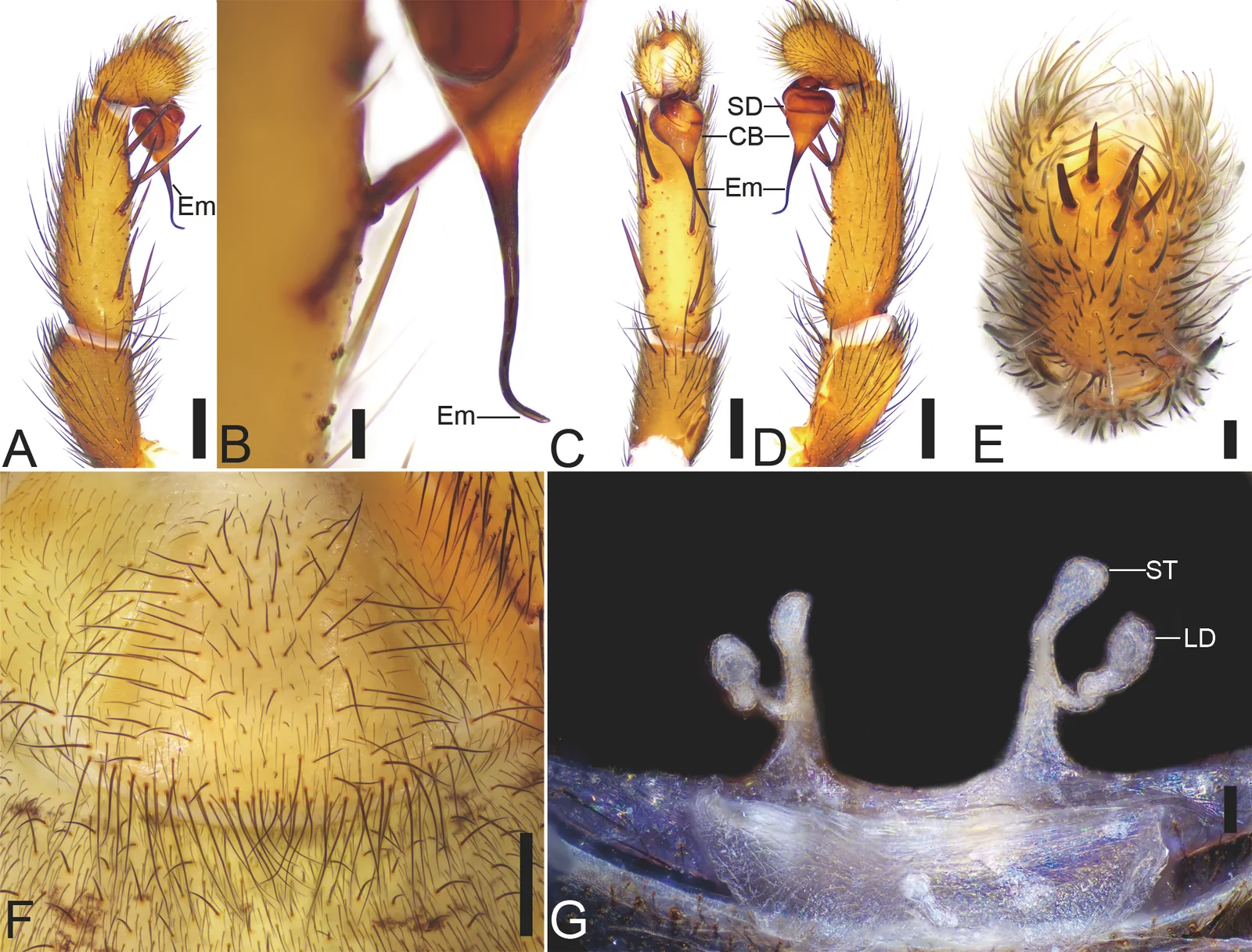
*Raveniola zhangzongxuni* sp. nov., **A**−**E**, male (holotype) and **F**, **G**, female (paratype). **A** palp, prolateral view; **B** psembolus, prolateral view; **C** palp, ventral view; **D** same, retrolateral view; **E** cymbium, dorsal view; **F** genital area, ventral view; **G** vulva, dorsal view. Abbreviations: CB − copulatory bulb, Em − embolus, LD − lateral diverticulum, SD − sperm duct, ST − spermathecal trunk. Scale bars: 0.5 mm (A, C, D, F), 0.1 mm (B, E, G).

**Figure 4.**
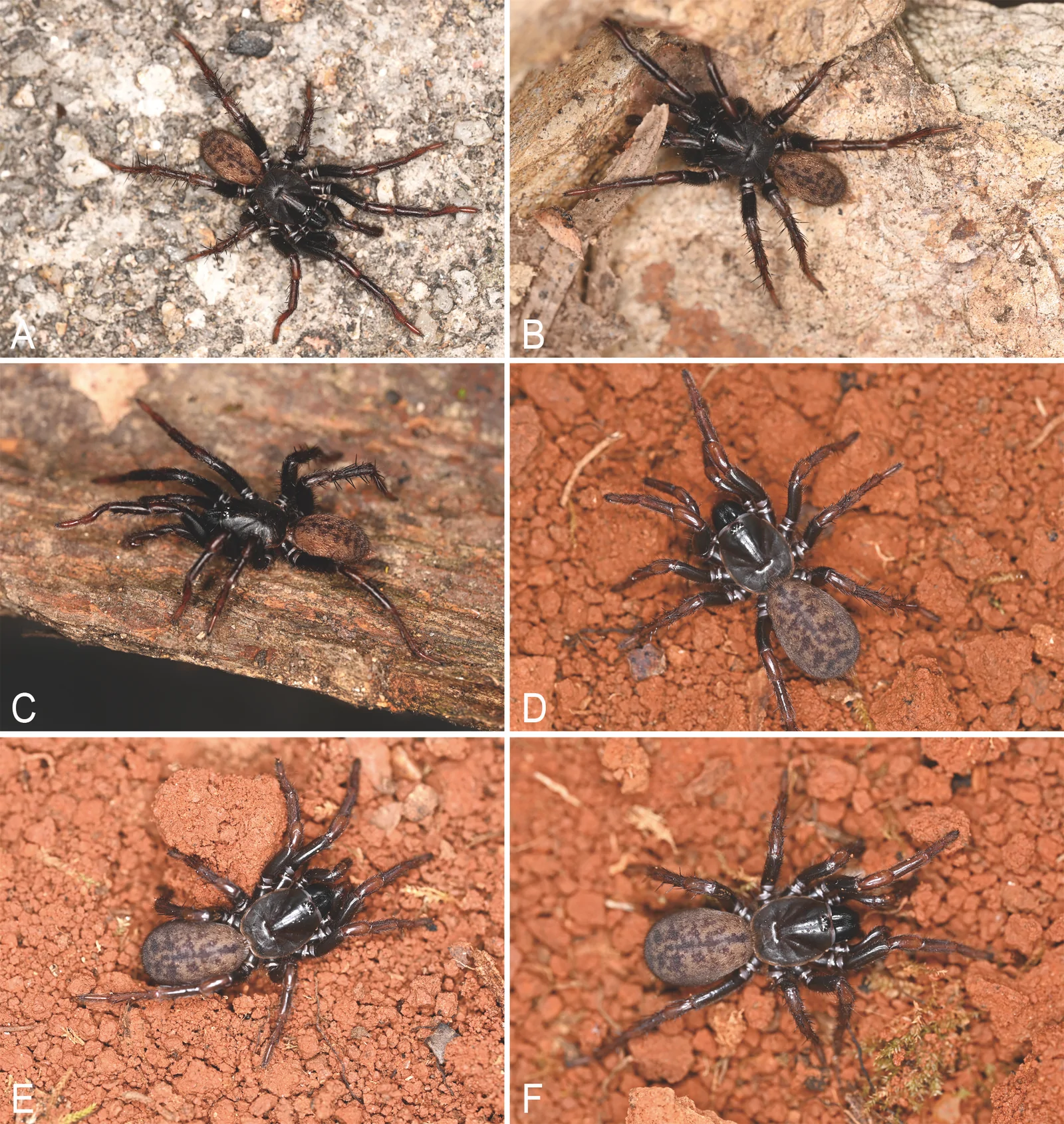
*Raveniola zhangzongxuni* sp. nov., photos of living specimen. **A−C** male; **D−F** female.

Spermathecae (Fig. 3F and G). Each of paired spermathecae Y-shaped with long spermathecal trunk carrying relatively long lateral diverticulum with curved median part and pronounced terminal fundus.

#### Diagnosis

Differs from *Raveniola chayi* Li & Zonstein, 2015 by having the dorsal abdominal pattern consisting of numerous darker spots on a lighter background (vs. numerous lighter spots on a darker background), the maxillae with 3 or 4 cuspules (vs. 12–15), the tibial length/cymbial length ca. 2.5 on male palp (vs. > 3.4), the copulatory bulb is relatively broader ca. 1.2–1.5 times as long as wide (vs. 2.5–4 times) and the lateral diverticulum arising from about 1/3 of spermathecal trunk (vs. about the base of spermathecal trunk). The new species also differs from *R. gracilis* Li & Zonstein, 2015 by the ventral abdominal pattern consisting of some darker spots on a lighter background (vs. pale yellow, without spots), the male PMS absent (vs. present), the maxillae with 3 or 4 cuspules (vs. 4–6), the tibial length/cymbial length ca. 2.5 on male palp (vs. > 2.6), the copulatory bulb is relatively broader ca. 1.2–1.5 times as long as wide (vs. nearly as long as wide) and the lateral diverticulum approximately 0.6 times as long as spermathecal trunk (vs. approximately 0.3 times).

#### Etymology

The species is named in honour of Zong-xun Zhang, one of the famous figures of the extremely hard and bitter struggle in Jinggangshan; noun (name) in genitive case.

#### Distribution

Known only from Jinggangshan City in Jiangxi Province, China.

#### Ecology

Female specimens were collected by digging from soil burrows, whereas male specimen was collected from beneath stones.

#### DNA barcode

ATGTAGACTTCTGGGTGACCAAAAAATCAAAATAAATGCTGAAACAAAATAGGATCCCCCCCTCCCGCTGGATCAAAAAAAGATGTATTAAAATTTCGATCAGTCAATAACATAGTAATAGCCCCCGCTAAAACAGGTAAAGATAATAGCAACAAAATAGCAGTAATAAGTACAGATCATACAAATAATGGGACCCGTTCTATAGTTATCCCATATCTTCGCATATTTACAATAGTAGAAATAAAATTAATAGCTCCCATAATAGAAGACCCCCCAGCCAAATGTAAAGAAAAAATAGCAAAATCTATCCCCCCACCTCTATGCCCCATACCAGAAGAAAGAGGAGGATAAATCGTTCACCCTGCCCCAACACCTCTGTCAGTCAATGACGATAAAACTAAAAAAAATAAAGAAGGTGGTAACAATCAAAATCTCAAATTATTTATCCGAGGAAACGCTATATCAGGAGCACCCAATATTAAAGGAATCAATCAGTTACCAAACCCCCCGATCATAATCGGTATAACTATAAAAAAAATCATAACTAAAGCATGAGCTGTTACAATAACATTATAAAGATGATCATCACCCAACATTCTTCCAACTTGCCCTAGTTCTGTTCGAATAATTACTCTTATAGCTGTCCCCACCATAGCTGATCACACTCCAAACAATAAATACAAAGTACCAATGTCCTTATGATTAGTAGAAAA (male holotype, ASM-JGSU-Ara6960, GenBank accession number: PZ818224).

TTTCTACTAATCATAAGGACATTGGTACTTTGTATTTATTGTTTGGAGTGTGATCAGCTATGGTGGGGACAGCTATAAGAGTAATTATTCGAACAGAACTAGGGCAAGTTGGAAGAATGTTGGGTGATGATCATCTTTATAATGTTATTGTAACAGCTCATGCTTTAGTTATGATTTTTTTTATAGTTATACCGATTATGATCGGGGGGTTTGGTAACTGATTGATTCCTTTAATATTGGGTGCTCCTGATATAGCGTTTCCTCGGATAAATAATTTGAGATTTTGATTGTTACCACCTTCTTTATTTTTTTTAGTTTTATCGTCATTGACTGACAGAGGTGTTGGGGCAGGGTGAACGATTTATCCTCCTCTTTCTTCTGGTATGGGGCATAGAGGTGGGGGGATAGATTTTGCTATTTTTTCTTTACATTTGGCTGGGGGGTCTTCTATTATGGGAGCTATTAATTTTATTTCTACTATTGTAAATATGCGAAGATATGGGATAACTATAGAACGGGTCCCATTATTTGTATGATCTGTACTTATTACTGCTATTTTGTTGCTATTATCTTTACCTGTTTTAGCGGGGGCTATTACTATGTTATTGACTGATCGAAATTTTAATACATCTTTTTTTGATCCAGCGGGAGGGGGGGATCCTATTTTGTTTCAGCATTTATTTTGATTCTTTGGTCACCCAGAAGTCTACAT (female, ASM-JGSU-Ara7438, GenBank accession number: PZ818225).

## Discussion

Based on the known distribution of the 74 species compiled above, the present discovery of *Raveniola* from Jiangxi Province significantly extends the known range of the genus in eastern China. Previously, the easternmost records of *Raveniola* in China were confined to Zhejiang, Fujian and northern Guangdong, with no records from the intervening provinces. The new species discovered in Jinggangshan National Nature Reserve, Jiangxi Province, connects the south-western and eastern distribution ranges of the genus in China, making the known distribution of *Raveniola* in China more continuous. According to the latest taxonomic study by Zonstein and Marusik (2025), all known Chinese *Raveniola* species have been preliminarily sorted into four distinct species groups (*R. alpina*, *R. beelzebub*, *R. gracilis*, *R. montana*) by the structural peculiarities of the male and female copulatory organs, the shape of the male tibia and metatarsus I or II, the number and arrangement of the maxillary or labial cuspules and the other structures. Only *R. xizangensis* cannot yet be definitively assigned to any established species group due to insufficient morphological data (Zonstein and Marusik 2025). This new species can be assigned to the *R. gracilis* species group, based on the hook-shaped distal embolus and the maxillae only with 3 or 4 cuspules. The present finding adds important distributional data for understanding the phylogenetic relationships and biogeographic patterns of the genus in the Oriental Region. Furthermore, this record also indicates that the mygalomorph fauna of central-eastern China remains significantly under-sampled; targeted surveys of overlooked microhabitats, such as soil burrows and under-stone retreats, are likely to yield additional undescribed species in this region.

## Supplementary Material

XML Treatment for Raveniola zhangzongxuni
